# Synthesis of Samarium OxysulfateSm_2_O_2_SO_4_ in the High-Temperature Oxidation Reaction and Its Structural, Thermal and Luminescent Properties

**DOI:** 10.3390/molecules25061330

**Published:** 2020-03-14

**Authors:** Yu. G. Denisenko, E. I. Sal’nikova, S. A. Basova, M. S. Molokeev, A. S. Krylov, A. S. Aleksandrovsky, A. S. Oreshonkov, V. V. Atuchin, S. S. Volkova, N. A. Khritokhin, O. V. Andreev

**Affiliations:** 1 Department of General and Special Chemistry, Industrial University of Tyumen, 625000 Tyumen, Russia; yu.g.denisenko@gmail.com; 2 Institute of Chemistry, Tyumen State University, 625003 Tyumen, Russia; elenasalnikova213@gmail.com (E.I.S.); basovasofia@yandex.ru (S.A.B.); kna@utmn.ru (N.A.K.); o.v.andreev@utmn.ru (O.V.A.); 3 Department of General Chemistry, Northen Trans-Ural Agricultural University, 625003 Tyumen, Russia; 4 Laboratory of Crystal Physics, Kirensky Institute of Physics, Federal Research Center KSC SB RAS, 660036 Krasnoyarsk, Russia; msmolokeev@mail.ru; 5 School of Engineering Physics and Radioelectronics, Siberian Federal University, 660041 Krasnoyarsk, Russia; 6 Department of Physics, Far Eastern State Transport University, Khabarovsk 680021, Russia; 7 Laboratory of Molecular Spectroscopy, Kirensky Institute of Physics Federal Research Center KSC SB RAS, 660036 Krasnoyarsk, Russia; shusy@iph.krasn.ru (A.S.K.); oreshonkov@iph.krasn.ru (A.S.O.); 8 Laboratory of Coherent Optics, Kirensky Institute of Physics Federal Research Center KSC SB RAS, 660036 Krasnoyarsk, Russia; aleksandrovsky@kirensky.ru; 9 Institute of Nanotechnology, Spectroscopy and Quantum Chemistry, Siberian Federal University, 660041 Krasnoyarsk, Russia; 10 School of Engineering and Construction, Siberian Federal University, 660041 Krasnoyarsk, Russia; 11 Laboratory of Optical Materials and Structures, Institute of Semiconductor Physics, SB RAS, 630090 Novosibirsk, Russia; 12 Laboratory of Semiconductor and Dielectric Materials, Novosibirsk State University, 630090 Novosibirsk, Russia; 13 Research and Development Department, Kemerovo State University, 650000 Kemerovo, Russia; 14 Research Resource Center “Natural Resource Management and Physico-Chemical Research”, Tyumen State University, 625003 Tyumen, Russia; svolkova2008@mail.ru; 15 Laboratory of the chemistry of rare earth compounds, Institute of Solid State Chemistry, UB RAS, 620137 Ekaterinburg, Russia

**Keywords:** samarium, oxysulfate, structure, luminescence, thermochemistry

## Abstract

The oxidation process of samariumoxysulfide was studied in the temperature range of 500–1000 °C. Our DTA investigation allowed for establishing the main thermodynamic (∆Hº_exp_ = −654.6 kJ/mol) and kinetic characteristics of the process (E_a_ = 244 kJ/mol, A = 2 × 10^10^). The enthalpy value of samarium oxysulfate (ΔHºf (Sm_2_O_2_SO_4(monocl)_) = −2294.0 kJ/mol) formation was calculated. The calculated process enthalpy value coincides with the value determined in the experiment. It was established that samarium oxysulfate crystallizes in the monoclinic symmetry class and its crystal structure belongs to space group C2/*c* with unit cell parameters *a* = 13.7442 (2), *b* = 4.20178 (4) and *c* = 8.16711 (8)Å, β = 107.224 (1)°, *V* = 450.498 (9)Å^3^, *Z* = 4. The main elements of the crystalline structure are obtained and the cation coordination environment is analyzed in detail. Vibrational spectroscopy methods confirmed the structural model adequacy. The Sm_2_O_2_SO_4_luminescence spectra exhibit three main bands easily assignable to the transitions from ^4^G_5/2_ state to ^6^H_5/2_, ^6^H_7/2_, and ^6^H_9/2_ multiplets.

## 1. Introduction

The compounds of rare-earth elements (REEs) with tetrahedral anions, possessing a set of rather valuable properties, have attracted the attention of researchers for recent years. In particular, rare earth oxysulfates are used as precursors for the production of REE_2_O_2_S compounds [1,2,3]. The materials containing oxysulfates are of practical importance as phosphorescent material components and they can be used in X-ray computed tomography and the detection of radioactive radiation [4,5,6,7]. The structural and chemical properties of REE_2_O_2_SO_4_oxysulfates make it possible to consider them as promising materials for the chemical adsorption and storage of gaseous oxygen [8,9,10,11]. Commonly, oxysulfates are formed upon the decomposition of REE compounds containing, at least, one sulfate group: REE_2_(SO_4_)_3_ [12,13,14,15], REE_2_(OH)_4_SO_4_ [16,17]. Oxysulfates can also be obtained by the decomposition of organic sulfonates of various structures [18]. A direct synthesis method consists of the temperature treatment of oxides in the atmosphere of sulfur oxide (IV) and oxygen [19].

Usually, lanthanide ions, due to forbidden electronic *f-f* transitions, are doping components in different materials and, in this form, they exhibit the properties of phosphors [20,21,22,23,24,25]. In many cases; however, the unobvious crystallographic positions of doping ions in such compounds induce certain difficulties in the observation of such materials [26,27]. Thermal decomposition methods are a convenient tool for producing compounds and materials with desired properties. As it is known from the reported results, the initial material granules, under certain conditions, are able to maintain the original shape and size in the thermal decomposition process [28,29,30]. At the same time, the compounds with the stoichiometric lanthanide ion content attract attention in order to find efficient luminescent materials with low concentration quenching and to investigate specific mechanisms of luminescence quenching in them [31,32,33,34,35,36,37,38,39,40]. At the same time, the consideration of lanthanide-containing materials cannot be restricted only by their luminescent properties. The possibility of using lanthanide compounds with simple and complex anions as paramagnetic, catalytic, scintillation and solid oxide-fuel materials are being increasingly investigated [41,42,43,44,45,46]. The present study is aimed at the samarium oxysulfatesynthesis in the high-temperature oxidative process and exploration of their structural, thermal and spectroscopic properties.

## 2. Results and Discussion

### 2.1. Dynamic Oxidation of Sm_2_O_2_S and Thermal Stability of Sm_2_O_2_SO_4_

**Figure 1:**
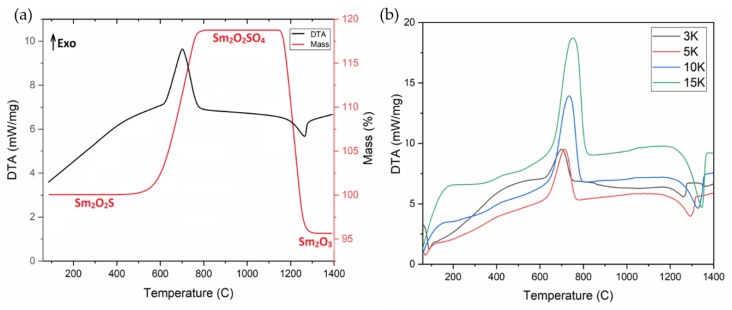
DTA/TGof Sm_2_O_2_S in synthetic air (**a**) and the shift of the peaks of thermal effects depending on the heating rate (**b**).

According to the differential thermal analysis (Figure 1a), the samarium oxysulfide oxidation begins at the temperature of 550 °C, proceeds in one stage and ends at 775 °C. The mass gain corresponds to the samarium oxysulfate (Sm_2_O_2_SO_4_) formation. The process is described by the reaction equation:

Sm_2_O_2_S + 2O_2_ → Sm_2_O_2_SO_4_ (1)

The resulting samarium oxysulfate is stable up to 1100 °C, and, then, it decomposes in one stage with the Sm_2_O_3_formation.The process can be described by the equation:

Sm_2_O_2_SO_4_ → Sm_2_O_3_ + SO_2_ + ^1^/_2_O_2_ (2)

The certain enthalpies of the two reactions allow us to write thermochemical equations:

Sm_2_O_2_S _(trig)_ + 2O_2(gas)_ → Sm_2_O_2_SO_4(monocl)_; ∆Hº = −654.6 kJ/mol (3)

Sm_2_O_2_SO_4(monocl)_ → Sm_2_O_3(cubic)_ + SO_2(gas)_ + ^1^/_2_O_2(gas)_; ∆Hº = 170.8 kJ/mol (4)

Using the data on the enthalpies of samarium oxide [47] and sulfur oxide (IV) [48] formation, the enthalpy of samarium oxysulfate formation was calculated by the Hess law and the value is equal to ∆Hº*_f_*(Sm_2_O_2_SO_4 (monocl)_) = −2294.0 kJ/mol.Substituting the enthalpy of Sm_2_O_2_SO_4_ formation in the equation for calculating the enthalpy of reaction 4 and using the samarium oxysulfide formation enthalpy ∆Hº*_f_*(Sm_2_O_2_S_(trig)_) = −1642.6 kJ/mol [49], we obtain the theoretical samarium oxysulfideoxidation enthalpy equal to −652.4 kJ/mol, which is perfectly compatible with the value determined according to the DTA measurements.

To study the kinetics of the Sm_2_O_2_SO_4_formation and decomposition processes, the thermal analysis of the samples was carried out at selected heating rates of 3, 5, 10, 15 °C/min (Figure 1b). Based on the DTA data at the pointed heating rates, the kinetic parameters of the processwere calculated.The temperature dependence of the oxidation rate of Sm_2_O_2_S to Sm_2_O_2_SO_4_ is characterized by relatively moderate parameters for such processes: E_a_ = 244 kJ/mol, A = 2 × 10^10^.The activation energy of the Sm_2_O_2_SO_4_decomposition to Sm_2_O_3_ is much higher and it is equalto 357 kJ/mol, but the preexponential factor is an order of magnitude lower and is equal to 1 × 10^9^. If we compare the parameters with those known for Eu_2_O_2_SO_4_ [14] (400 kJ/mol and 1 × 10^12^, respectively), this corresponds to wider peaks in the DTA curves for the Eu_2_O_2_SO_4_decomposition, which indicates its higher kinetic stability, as compared to that of Sm_2_O_2_SO_4_. In addition, the significantly higher preexponential factor for the Eu_2_O_2_SO_4_decomposition, in comparison with that of Sm_2_O_2_SO_4_, suggests that the Sm_2_O_2_SO_4_ symmetry is, at least, not higher than that of Eu_2_O_2_SO_4_. The reduced kinetic stability of Sm_2_O_2_SO_4_, in comparison with that of Eu_2_O_2_SO_4_, is in a good agreement with the enthalpy values of compound decomposition.

### 2.2. Isothermal Oxidation of Sm_2_O_2_S

**Figure 2:**
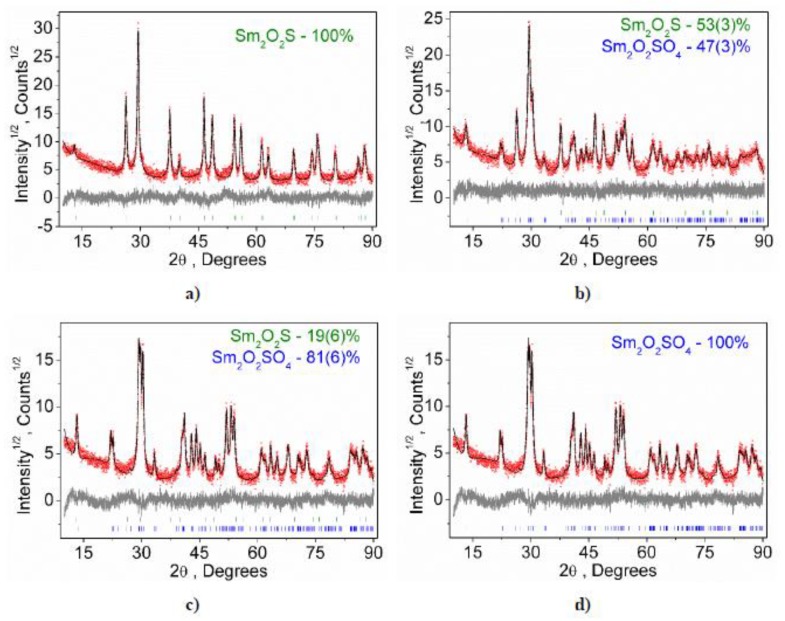
X-ray diffraction patterns of Sm_2_O_2_S (**a**) and the samples subjected to oxidation at 600 °C for 2 h (**b**), 5 h (**c**) and 7 h (**d**).

At the temperature of 500 °C for 10 h, according to the results of X-ray phase analysis, there is no phase composition change of the Sm_2_O_2_S sample (Figure 2a). However, starting from 600 °C, the phase composition of the sample changes rapidly and, after only two hours, approximatelyhalf of Sm_2_O_2_S enters into the reaction (Figure 2b). After five hours, only about 20% of samarium oxysulfide remains in the sample (Figure 2c). In 7 h of the process, the sample contains only pure samarium oxysulfate (Figure 2d). The temperature increase to 700 °C leads to a sharp increase in the reaction rate, and the complete oxidation of the sample is reached for one hour. Such behavior differs significantly from the EuS oxidation process [35] where such pronounced rate temperature dependence is not observed. This effect is obviously related to the fact that only one reaction occurs during the Sm_2_O_2_S oxidation, in contrast to the EuS oxidation process, in which several parallel competing processes are realized. The samarium oxysulfide samples oxidation at 800, 900 and 1000 °C leads to the production of Sm_2_O_2_SO_4_ samples for one hour. An increase in the exposure time at these temperatures does not lead to a further change in the phase composition of the samples.

**Figure 3:**
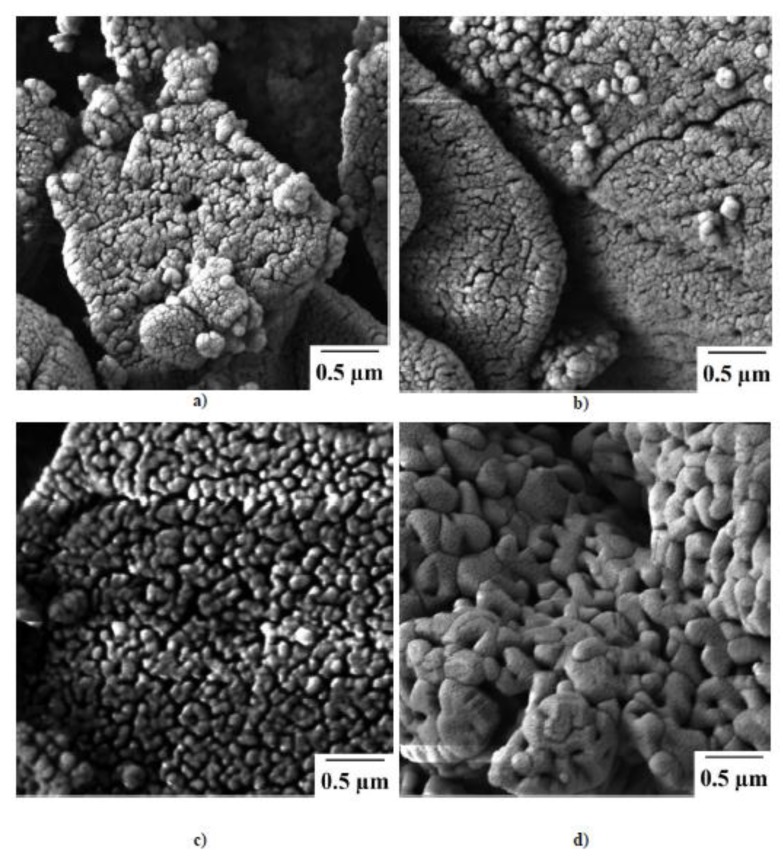
SEM images of Sm_2_O_2_S (**a**) and of Sm_2_O_2_SO_4_ samples obtained at temperatures of 600 °C (**b**), 800 °C (**c**) and 1000 °C (**d**).

According to scanning electron microscopy, the samarium oxysulfide powder is formed by agglomerates sized 2–3 μm. The agglomerates have a clear granule structure. The initial granules have a size of about 50–100 nm (Figure 3a). Carrying out the oxidation process at 600 °C practically does not affect the change in the microstructure of the obtained Sm_2_O_2_SO_4_ samples (Figure 3b). A further increase in the process temperature leads to the agglomeration of the initial granules while maintaining the overall structure of the agglomerates (Figure 3c,d). In the Sm_2_O_2_SO_4_ sample obtained at 1000 °C, the initial granules have sizes from 250 nm to 0.5 μm. It should be pointed that the particlemicrostructure preservation is an important effect determining the possibility of applying the oxidation process to the synthesis of biocompatible materials based on rare earth oxysulfates [28,29,30].

**Figure 4:**
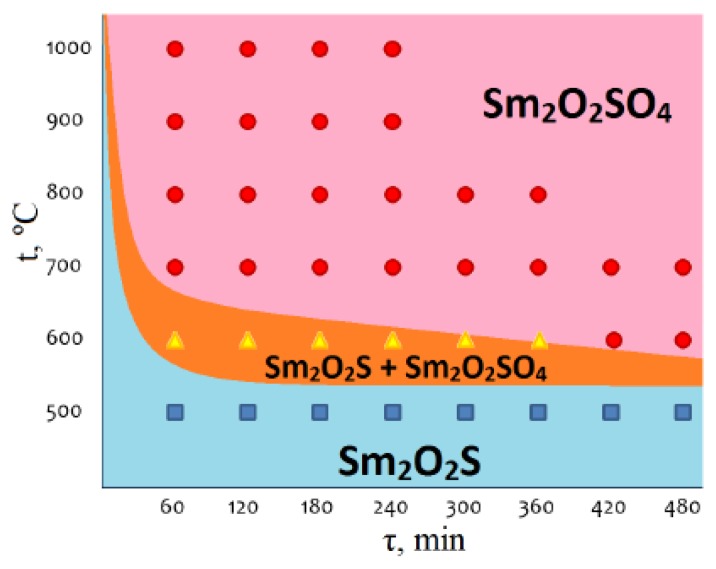
Kinetic scheme of changes in the chemical composition during the samarium oxysulfide oxidation.

Based on the analysis of available experimental data on the phase composition of the samples obtained in isothermal processes, a kinetic diagram was built for the chemical composition changes during the samarium oxysulfide oxidation with air oxygen (Figure 4). In the diagram, threephase statefields can be observed. Two single-phase fields related to the stability conditions for compounds Sm_2_O_2_S (blue) and Sm_2_O_2_SO_4_ (pink), and the intermediate two-phase field of Sm_2_O_2_S + Sm_2_O_2_SO_4_ (orange), whichboundaries are clearly governed by the thermodynamic and kinetic parameters of the process, are determined. As it is seen, the pure Sm_2_O_2_SO_4_ phase can be synthesized at temperatures ≥700 °C for the reaction time 60–480 min. The phase fieldposition in the diagram allows one to determine the conditions for the targeted preparation of the samples with specified phase compositions.

### 2.3. Structural Properties of Sm_2_O_2_SO_4_

**Figure 5:**
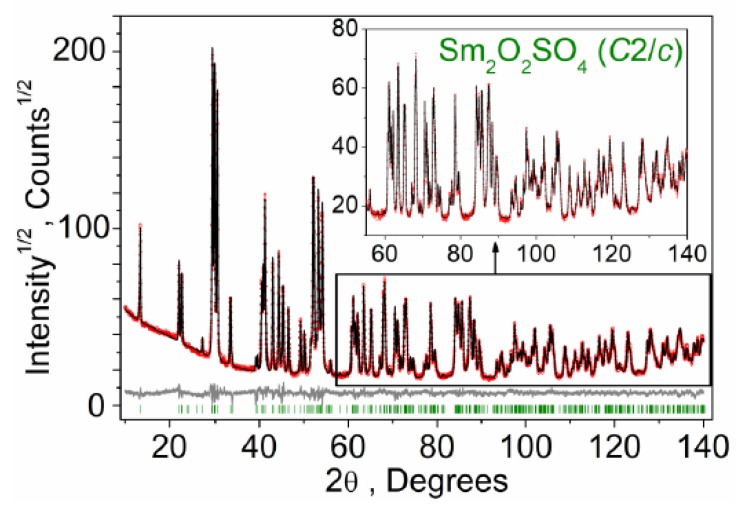
Difference Rietveld plot of Sm_2_O_2_SO_4_.

A sample of Sm_2_O_2_SO_4_ for structural analysis was obtained by oxidizing samarium oxysulfide in the air at 900 °C for 10 h. The Rietveld refinement was carried out by using TOPAS 4.2 [50] which accounts the esd’s of each point by a special weight scheme. All peaks were indexed by a monoclinic cell (*C*2*/c*) with the parameters close to those of Eu_2_O_2_SO_4_ [35] and; therefore, the crystal structure of Eu_2_O_2_SO_4_was taken as a starting model for Rietveld refinement. The Eu^3+^ site in the Eu_2_O_2_SO_4_ structure was considered as occupied by the Sm^3+^ ion. In order to reduce the number of refined parameters, only one thermal parameter was refined for all O atoms. The refinement was stable and gave low *R*-factors (Table 1, Figure 5). The atom coordinates and main bond lengths obtained in Sm_2_O_2_SO_4_aresummarized in Table 2 and Table 3, respectively. The cif and checkcif files are given in Supplementary Materials. The crystallographic data are deposited in the Cambridge Crystallographic Data Centre (CSD # 1968636). The data can be downloaded from the site (www.ccdc.cam.ac.uk/data_request/cif).

The main difference of Eu_2_O_2_SO_4_ and Sm_2_O_2_SO_4_ structures is observed in their cell parameters and cell volumes. The former crystal has *a* = 13.65826(27), *b* = 4.188744(73), *c* = 8.14400(14) Å, β = 107.2819(21)°, *V* = 444.892(15) Å^3^, and the compound under investigation Sm_2_O_2_SO_4_ is characterized by *a* = 13.7442 (2), *b* = 4.20178 (4), *c* = 8.16711 (8) Å, β = 107.224 (1)°, *V* = 450.498 (9) Å^3^. It is clearly seen that the cell parameters and cell volume of Eu_2_O_2_SO_4_ are smaller than those of Sm_2_O_2_SO_4_, and it is consistent with the fact that ion radius IR(Eu, CN=9) = 1.12 Å is smaller than IR(Sm, CN=9) = 1.132 Å.

**Figure 6:**
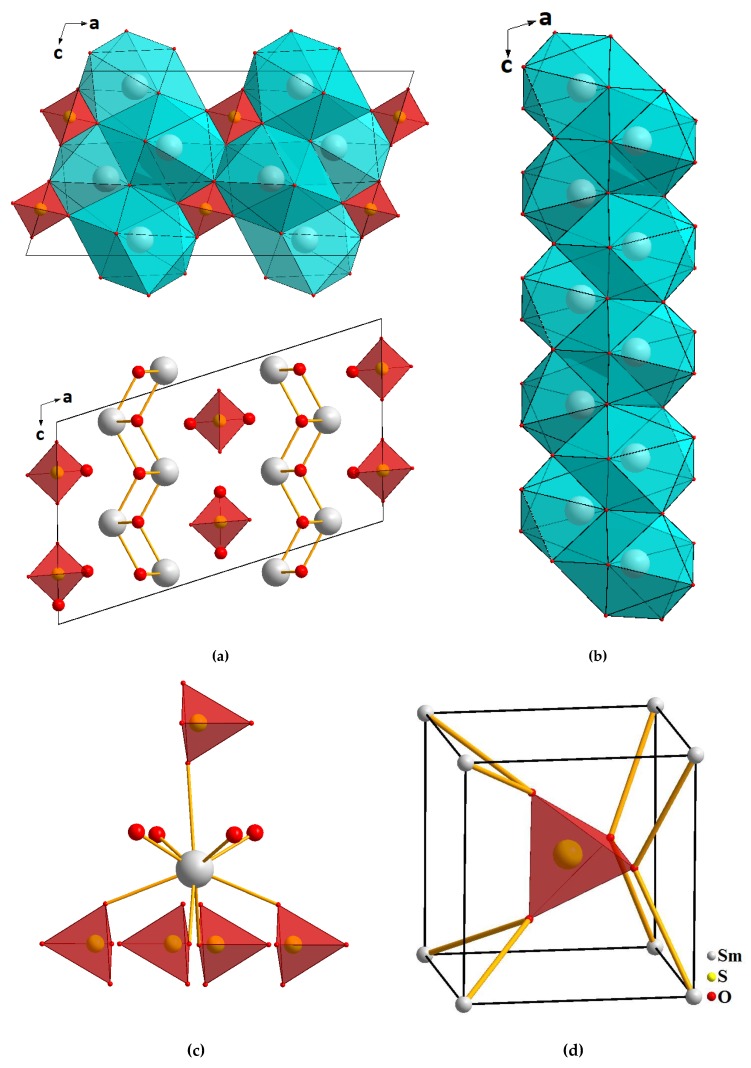
Projections of the Sm_2_O_2_SO_4_ crystal structure (**a**), the structure of zigzag chains [SmO_9_]_n_ (**b**), coordination of samarium (**c**) and coordination of sulfate tetrahedra (**d**) in the structure.

**Figure 7:**
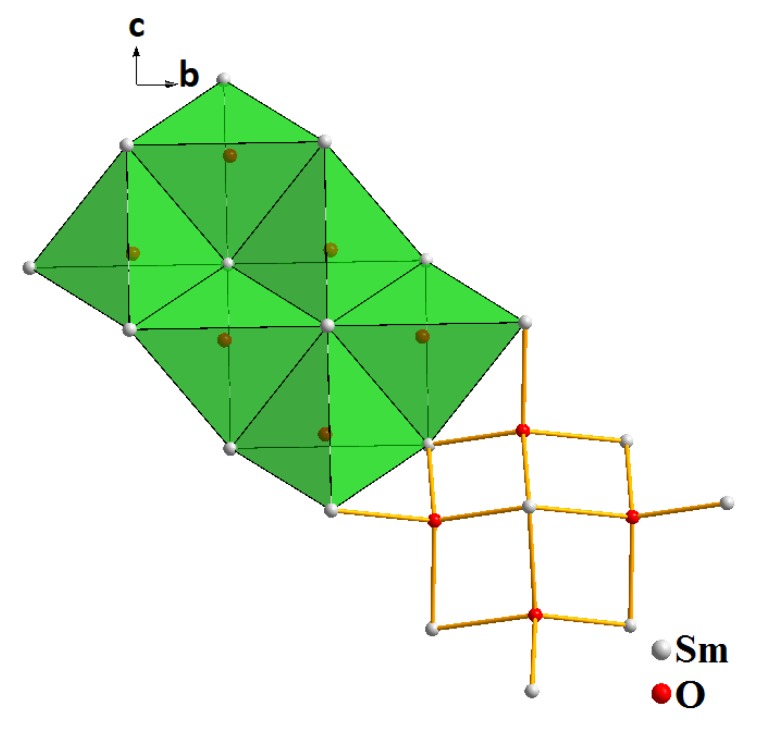
The cationic layers structure formed by the junction of tetrahedra [OSm_4_].

As shown in Figure 6, the structure is represented by the alternation of cationic layers [Sm_2_O_2_^2+^]_n_ with the anionic layers consisting of isolated [SO_4_]^2−^ tetrahedra. Both layers are parallel to (100) (Figure 6a). All samarium atoms occupy identical crystallographic positions and are coordinated by nine oxygen atoms: five oxygen atoms belong to monodentate-bound sulfate groups, and the remaining oxygen atoms are bridging (Figure 6c). Thus, the samarium atom in the structure forms a coordination environment shaped as a three-cap trigonal prism. Two caps of the coordination polyhedron, connected along the edge at the angle of 180°, form a plane of four oxygen atoms. The trigonal prism and caps in the coordination polyhedron are deformed due to the difference in the Sm-O bond lengths. One Sm-O bond is much longer than the others. As a result, the coordination number of samarium is classified as 8 + 1. The SmO_9_ polyhedra join with each other forming an infinite chain along the *c*-axis (Figure 6b). The oxygen atoms of SO_4_ groups are coordinated by sulfur and samarium atoms. The sulfate tetrahedron is surrounded by eight samarium atoms, resulting in the formation of sphere-shaped coordination as almost a perfect cube (Figure 6d). Each bridging oxygen atom is coordinated by four samarium atoms, and it results in the formation of [OSm_4_] tetrahedra. These tetrahedra, sequentially pair wise connected with each other, form unlimited zigzag chains. The interconnected chains form continuous layers (Figure 7).

### 2.4. Vibrational Spectra of Sm_2_O_2_SO_4_

**Figure 8:**
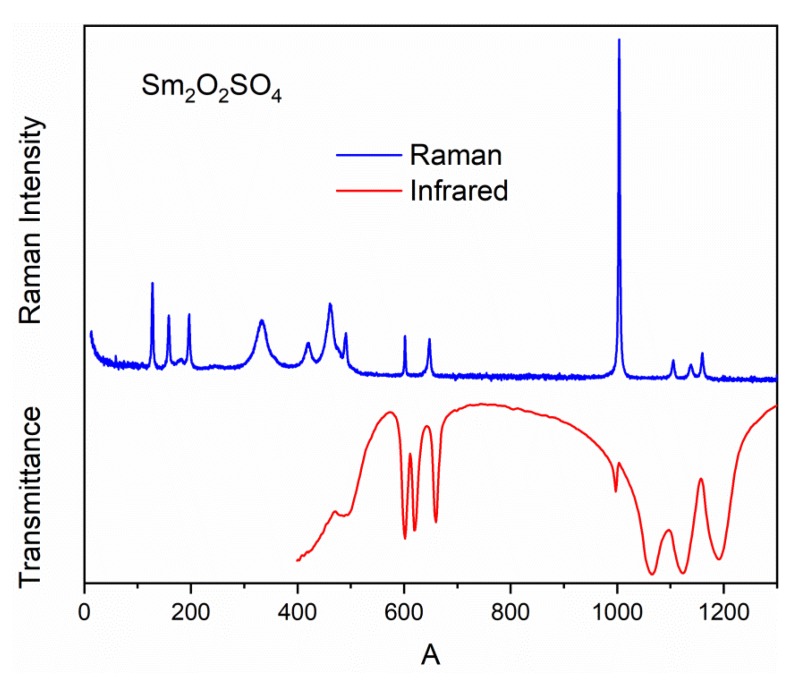
Raman and Infrared spectra of Sm_2_O_2_SO_4_ powder.

Raman and Infrared spectra of Sm_2_O_2_SO_4_ are shown in Figure 8. The irreducible vibrational representations for the monoclinic structure of Sm_2_O_2_SO_4_ at the center of the Brillouin zone is Γ_vibr_ = 13*A*_g_ + 13*A*_u_ + 14*B*_g_ + 14*B*_u_, where *A*_u_ + 2*B*_u_ are acoustic modes and 13*A*_g_ + 14*B*_g_ are Raman-active modes, while the 12*A*_u_ + 12*B*_u_ modes are active in IR spectra. The free tetrahedral [SO_4_]^2−^ ion of the *T*_d_ symmetry exhibits four internal vibrations. All four vibrations are Raman-active, whereas only ν_3_ and ν_4_ are Infrared-active. In the solid state, ν_3_ and ν_4_ may split into two or three bands because of the site effect [51]. The correlation diagram of internal vibrations between the free [SO_4_]^2−^ ions of the *T*_d_symmetry, its site symmetry (*C*_2_) and the factor group symmetry (*C*_2h_) of a unit cell is given in Table 4.

From the correlation diagram, we can conclude that four spectral bands should be observed in the range of stretching vibrations of the SO_4_ tetrahedra (975–1225 cm^−1^) in the Raman spectrum. The IR spectrum of the Sm_2_O_2_SO_4_ structure should contain four bands in the range of stretching vibrations of [SO_4_]^2−^ ions, too. Three of them are ν_3_ antisymmetric stretching and one is related to ν_1_ symmetric stretching vibration. The ν_4_ bending vibrations locate in the range of 575–675 cm^−1^. The relevant spectral bands can be seen in Figure S1 (Supplementary Materials) and Figure 8. The Raman bands associated with the ν_4_ bending vibrations of SO_4_tetrahedra are overlapped with bands related to Sm-O vibrations, and these vibrations locate in the range of 300–500 cm^−1^. The low-intensity bands in Raman spectra around 250 cm^−1^ should correspond to rotational vibrations of [SO_4_]^2−^ ions [52]. The remaining spectral bands below 200 cm^-1^ are translational vibrations of SmO_9_polyhedra, SO_4_tetrahedra and Sm^3+^ ions.

### 2.5. Luminescent Properties of Sm_2_O_2_SO_4_

**Figure 9:**
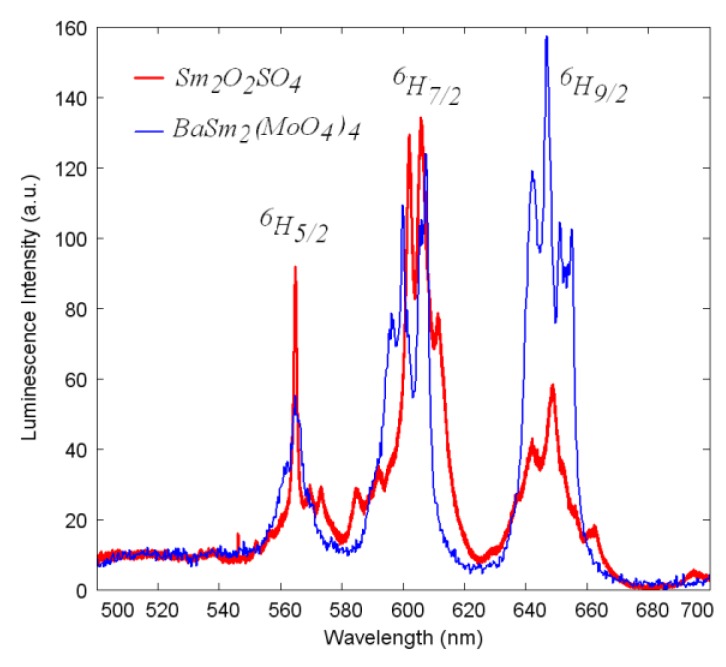
The luminescence spectra of Sm_2_O_2_SO_4_ (red) and of the reference crystal (BaSm_2_(MoO_4_)_4_, blue). For better comparison, the reference crystal spectrum is divided by 10.

The Sm_2_O_2_SO_4_ luminescence spectrum was recorded using the excitation by theGaN laser diode with the central wavelength 410 nm (24400 cm^−1^) falling into three closely-spaced Sm^3+^ transitions from the ground state ^6^H_5/2_ to ^6^P_5/2_, ^4^M_19/2_ and ^4^L_13/2_ excited states. The obtained spectrum is presented in Figure 9 in comparison with the luminescence spectrum of another highly-concentrated samarium-containing BaSm_2_(MoO_4_)_4_ crystal [39]. The structure of luminescence spectra of both Sm_2_O_2_SO_4_ and the reference crystal is rather similar and exhibits three main bands easily assignable to the transitions from the^4^G_5/2_ state to ^6^H_5/2_, ^6^H_7/2_ and ^6^H_9/2_multiplets. However, the distribution of the intensities between three mentioned channels in Sm_2_O_2_SO_4_ is slightly different from that of the reference crystal, while the red transition to the^6^H_9/2_ state dominates in the reference crystal, the orange transition to the ^6^H_7/2_ state prevails in Sm_2_O_2_SO_4_. This difference demonstrates the possibility of controlling the samarium ion emission chromaticity via the crystal field engineering that allows certain variation of Judd–Ofelt intensity parameters. We must note that the reference crystal spectrum was divided by 10 for a better comparison of the shapes. Therefore, we must deduce that concentration quenching of the luminescence in Sm_2_O_2_SO_4_ is rather high in comparison with (e.g., molybdate crystalline lattices).

## 3. Materials and Methods

### 3.1. Synthesis Methods

Samarium oxysulfide was obtained by the reduction of samarium sulfate Sm_2_(SO_4_)_3_ (99.9%, Merck Ltd., Germany) in the hydrogen atmosphere at the temperature of 700 °C. The installation scheme for carrying out the high-temperature recovery processes is shown in Figure S2 (Supplementary Materials). High-purity hydrogen was obtained by the electrolytic method in a SPECTR-6M hydrogen generator (Spectr, Moscow, Russia). The temperature control and regulation were carried out using a microprocessor controller (Thermoceramics, Moscow, Russia). The temperature measurement in the reaction zone was provided by a chromel–alumel thermocouple. A weighed amount of dry Sm_2_(SO_4_)_3_ was placed in a quartz reactor, and it was purged with hydrogen from the generator for 30 min at the rate of 6 L/h. After that, the reactor was placed in a heated vertical furnace and kept for 5 h. After the completion of the recovery process, the reactor was removed from the furnace and cooled to room temperature. The process proceeding during the recovery is described by the equation:

Sm_2_(SO_4_)_3_ + 12H_2_ → Sm_2_O_2_S + 2H_2_S↑ + 10H_2_O↑ (5)

To study the samarium oxysulfide oxidation with air oxygen, 0.5 g of Sm_2_O_2_S sample was uniformly distributed as a thin layer over a ceramic boat bottom with the area of 3 × 5 cm^2^. In order to prevent the tight layer formation during the oxidation process, all samarium oxysulfide samples were crushed in an agate mortar with acetone addition. After the filling, the ceramic boat was placed in a horizontal furnace (Thermoceramics, Moscow, Russia) heated to the required temperature and the processing was carried out in a continuous air flow. After the required time, the boat was removed from the oven and cooled to room temperature in a desiccator with the silica gel to avoid surface hydration. A study of the phase composition of obtained oxidized sample was carried out by the X-ray diffraction method. The isothermal oxidation experiments were carried out at the temperatures of 500, 600, 700, 800, 900 and 1000 °C. The total time of the oxidation process at each temperature did not exceed 10 h.

### 3.2. Methods of Physico-Chemical Analysis

The thermal analysis in the synthetic air (80% Ar-20% O_2_) flow was carried out on a Simultaneous Thermal Analysis (STA) equipment 499 F5 Jupiter NETZSCH (Netzsch, Selb, Germany). The powder samples were inserted into alumina crucibles. The heating rate was 3 °C/min. For the enthalpy determination, the equipment was calibrated with the use of standard metal substances, such as In, Sn, Bi, Zn, Al, Ag, Au and Ni. The heat effect peaks were determined with the package «Proteus 6 2012» (Netzsch, Selb, Germany). The peak temperature and area in parallel experiments were reproduced at an inaccuracy lower than 3%. The kinetic parameters determination was based on Kissinger formula [53] in the linearized form:

(6) $$\frac{1}{T}=\frac{R}{E}ln\frac{AR}{E}-\frac{1}{E}Rln\frac{b}{T^{2}}$$

where *T* is the temperature with a maximum reaction rate; *b*—heating rate; *E*—activation energy and *A*—preexponential factor. The representative examples of using the formula in topochemical processes can be found elsewhere [54,55,56].

To determine the phase composition of the samples at various oxidation stages, we used a BRUKER D2 PHASER X-ray diffractometer (Bruker, Billerica, MA, USA) with a linear detector LYNXEYE (CuKα radiation, Ni-filter, Bruker, Billerica, MA, USA). The crystal structure was refined using the Rietveld method in the TOPAS 4.2 program [50]. The powder diffraction data of Sm_2_O_2_SO_4_ for Rietveld analysis were collected at room temperature with a Bruker D8 ADVANCE powder diffractometer (Cu-Kα radiation, Bruker, USA) equipped with a linear detector VANTEC (Bruker, Billerica, MA, USA). The step size of 2θ was 0.016°, and the counting time was 5 s per step. The particle morphology analysis was carried out on an electron microscope JEOL JSM-6510LV (Japan). The X-ray energy-dispersive analyzer (Oxford Instruments, Abington, UK) was used to register the X-ray signal at recording the element spectrum in the selected regions of the sample surface. The possible inaccuracy of elemental content determination by this method was equal to ±0.2%. The Fourier-transform infrared spectroscopy (FTIR) analysis was carried out with the use of Fourier-Transform Infrared Spectrometer FSM 1201 (Infraspec, Moscow, Russia). The sample for the investigation was prepared in the tablet shape with the addition of annealed KBr. The Raman scattering spectra of Sm_2_O_2_SO_4_ were collected in backscattering geometry, using a triple monochromator Horiba JobinYvon T64000 Raman spectrometer (JobinYvon, France) operating in subtractive mode. The spectral resolution for the recorded Stokes side Raman spectra was about 1 cm^−1^ (this resolution was achieved by using gratings with 1800 grooves mm^−1^ and 100 micrometer slits). Single-mode krypton 647.1 nm of Lexel Kr^+^ laser of 3 mW on the sample was used as an excitation light source. The luminescence spectra at room temperature were recorded using a Horiba-Jobin-Yvon T64000 spectrometer (JobinYvon, France) and GaN laser diode with the central wavelength 410 nm. Spectral resolution of the measurement channel of the spectrometer was 2.7 cm^−1^.

## 4. Conclusions

A comprehensive study of the samariumoxysulfide oxidation process was carried out. The kinetic and thermodynamic characteristics of the process were established. The effect of oxidation temperature on the morphology of samarium oxysulfate samples was evaluated. The main structural and spectroscopic characteristics of samarium oxysulfatewere determined. According to the X-ray powder diffraction data, the monoclinic symmetrywasestablished.The main structural elements and their influence on the properties of the compound were analyzed. The theoretical calculations of vibration spectra confirm the adequacy of the structural model, which is important for such complex structures with the ambiguity in the choice of the structural model. The Sm_2_O_2_SO_4_luminescent-spectral characteristics were determined. The luminescence spectrum consists of three main luminescent bands originating from the ^4^G_5/2_ state, the transition to ^6^H_7/2_ in the orange part of the spectrum being dominant.

## Figures and Tables

**Table 1:** Main parameters of processing and refinement of the Sm_2_O_2_SO_4_ sample.

Compound	Sm_2_O_2_SO_4_
Space group	*C*2/*c*
*a*, Å	13.7442 (2)
*b*, Å	4.20178 (4)
*c*, Å	8.16711 (8)
*β*, º	107.224 (1)
*V*, Å^3^	450.498 (9)
*Z*	4
*2θ*-interval, º	10–140
*R_wp_*, %	6.16
*R_p_*, %	4.52
*R_exp_*, %	2.78
*χ* ^2^	2.22
*R_B_*, %	1.70

*a*, *b*, *c* and β—cell parameters; *V*—cell volume, *Z*—number of formula in unit cell; *R*_wp_—weighted profile *R*-factor, *R*_p_—profile *R*-factor; *R*_exp_—expected *R*-factor; *χ*^2^—goodness of fit, *R*_B_—Bragg *R*-factor.

**Table 2:** Fractional atomic coordinates and isotropic displacement parameters (Å^2^) of Sm_2_O_2_SO_4_.

	*x*	*y*	*z*	*B*_iso_
Sm1	0.16930 (3)	0.5015 (4)	0.0850 (3)	0.45 (2)
S1	0	0.0339 (15)	0.25	1.63 (8)
O3	0.0904 (4)	0.8717 (12)	0.2840 (19)	0.75 (7)
O2	0.9996 (8)	0.2711 (12)	0.0985 (8)	0.75 (7)
O1	0.2474 (3)	0.022 (2)	0.120 (3)	0.75 (7)

*B*_iso_—isotropic thermal parameter.

**Table 3:** Main bond lengths (Å) of Sm_2_O_2_SO_4_.

Sm1-O3	2.698 (9)	Sm1-O1 ^v^	2.417 (10)
Sm1-O3 ^i^	2.846 (13)	Sm1-O1 ^vi^	2.291 (14)
Sm1-O3 ^ii^	3.202 (3)	Sm1-O1 ^vii^	2.346 (19)
Sm1-O2 ^iii^	2.558 (9)	S1-O3 ^viii^	1.372 (5)
Sm1-O2 ^iv^	2.547 (8)	S1-O2 ^iii^	1.588 (7)
Sm1-O1	2.259 (9)		

Symmetry codes: (i) *x*, -*y*+1, *z*-1/2; (ii) 1/2-x, -1/2+y, 1/2-z; (iii) *x*-1, *y*, *z*; (iv) -*x*+1, -*y*+1, -*z*; (v) *x*, *y*+1, *z*; (vi) -*x*+1/2, -*y*+1/2, -*z*; (vii) -*x*+1/2, *y*+1/2, -*z*+1/2; (viii) *x*, *y*-1, *z.*

**Table 4:** Correlation diagram of internal vibrations of the [SO_4_]^2−^ ions in the Sm_2_O_2_SO_4._

Wavenumber (cm^−1^) [51]	*T*_d_ Point Group	*C*_2_ Site Symmetry	*C*_2h_ Factor Group Symmetry
983	*A*_1_ (ν_1_)	*A*	*A*_g_ *+ A*_u_
450	*E* (ν_2_)	2*A*	2*A*_g_ + 2*A*_u_
1105	*E* (ν_3_)	*A* + 2*B*	*A*_g_* + A*_u_+ 2*B*_g_+ 2*B*_u_
611	*E* (ν_4_)	*A*+ 2*B*	*A*_g_*+ A*_u_+ 2*B*_g_+ 2*B*_u_

## Supplementary Materials

The following are available online. The cif and checkcif files. Figure S1: Decomposition of the Sm_2_O_2_SO_4_ Raman spectrum in the range of ν_4_ vibrations of [SO_4_]^2−^ ions, Figure S2: Installation scheme of processing substances in a stream of hydrogen: 1—a hydrogen generator; 2—power control unit of electricity supplied to the furnace; 3—Thermocouple; 4—electric heating furnace; 5—reactor with the processed substance.
