# Supplementary material for: Synthesis of Samarium OxysulfateSm2O2SO4 in the High-Temperature Oxidation Reaction and Its Structural, Thermal and Luminescent Properties
Source: Molecules. 2020 Mar 14;25(6):1330. doi: 10.3390/molecules25061330 (PMC7144115; doi:10.3390/molecules25061330)
Supplement: Supplementary file 1 [file molecules-25-01330-s001.zip › molecules-725098 supplementary.pdf]

molecules

Article

Synthesis of Samarium Oxysulfate$Sm_2O_2SO_4$ in the High-Temperature Oxidation Reaction and Its Structural, Thermal and Luminescent Properties

Yu.G. Denisenko [1], E.I. Sal'nikova [2,3], S.A. Basova [2], M.S. Molokeev [4,5,6], A.S. Krylov [7], A.S. Aleksandrovsky [8,9], A.S. Oreshonkov [7,10], V.V. Atuchin [11,12,13,*], S.S. Volkova [14], N.A. Khritokhin [2] and O.V. Andreev [2,15]

[1] Department of General and Special Chemistry, Industrial University of Tyumen, Tyumen 625000, Russia; yu.g.denisenko@gmail.com
[2] Institute of Chemistry, Tyumen State University, Tyumen 625003, Russia; elenasalnikova213@gmail.com (E.I.S.); basovasofia@yandex.ru (S.A.B.); kna@utmn.ru (N.A.K.); o.v.andreev@utmn.ru (O.V.A.)
[3] Department of General Chemistry, Northen Trans-Ural Agricultural University, Tyumen 625003, Russia
[4] Laboratory of Crystal Physics, Kirensky Institute of Physics, Federal Research Center KSC SB RAS, Krasnoyarsk 660036, Russia; msmolokeev@mail.ru
[5] School of Engineering Physics and Radioelectronics, Siberian Federal University, Krasnoyarsk 660041, Russia
[6] Department of Physics, Far Eastern State Transport University, Khabarovsk 680021, Russia
[7] Laboratory of Molecular Spectroscopy, Kirensky Institute of Physics Federal Research Center KSC SB RAS, Krasnoyarsk 660036, Russia; shusy@iph.krasn.ru (A.S.K.); oreshonkov@iph.krasn.ru (A.S.O.)
[8] Laboratory of Coherent Optics, Kirensky Institute of Physics Federal Research Center KSC SB RAS, Krasnoyarsk 660036, Russia; aleksandrovsky@kirensky.ru
[9] Institute of Nanotechnology, Spectroscopy and Quantum Chemistry, Siberian Federal University, Krasnoyarsk 660041, Russia
[10] School of Engineering and Construction, Siberian Federal University, Krasnoyarsk 660041, Russia
[11] Laboratory of Optical Materials and Structures, Institute of Semiconductor Physics, SB RAS, Novosibirsk 630090, Russia
[12] Laboratory of Semiconductor and Dielectric Materials, Novosibirsk State University, Novosibirsk 630090, Russia
[13] Research and Development Department, Kemerovo State University, Kemerovo 650000, Russia
[14] Research Resource Center "Natural Resource Management and Physico-Chemical Research", Tyumen State University, Tyumen 625003, Russia; svolkova2008@mail.ru
[15] Laboratory of the chemistry of rare earth compounds, Institute of Solid State Chemistry, UB RAS, Ekaterinburg 620137, Russia
* Correspondence: atuchin@isp.nsc.ru

Received: 5 February 2020; Accepted: 10 March 2020; Published: 14 March 2020

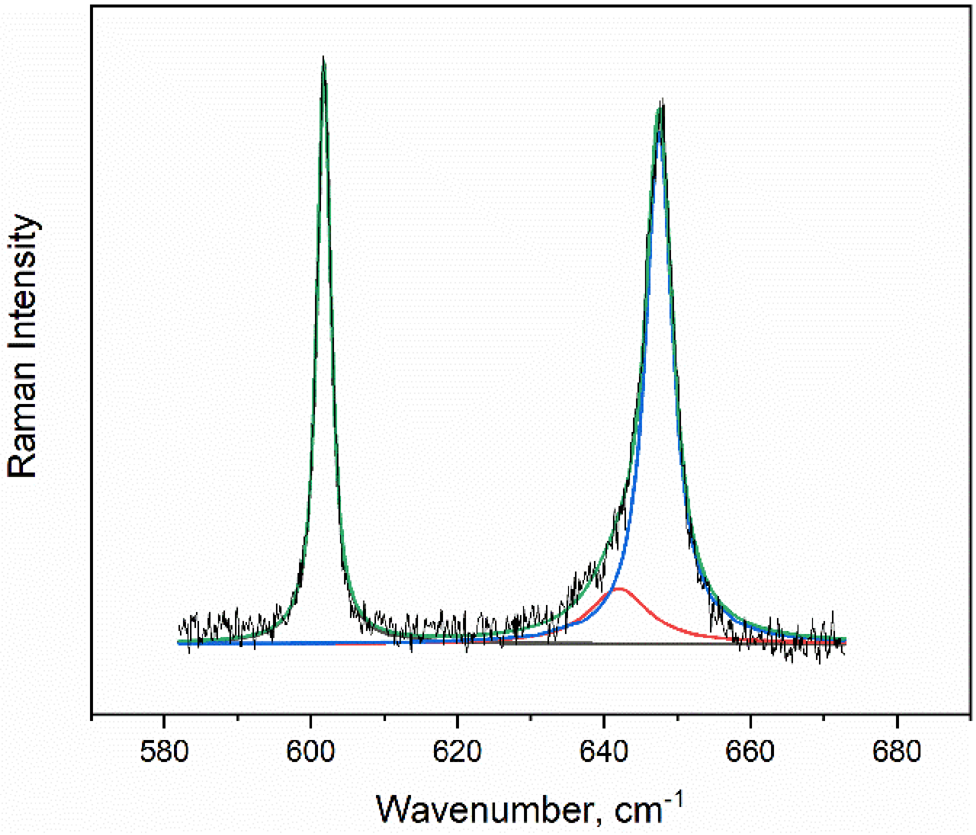

Figure S1. Decomposition of the $Sm_2O_2SO_4$ Raman spectrum in the range of ν_4 vibrations of $[SO_4]^{2-}$ ions.

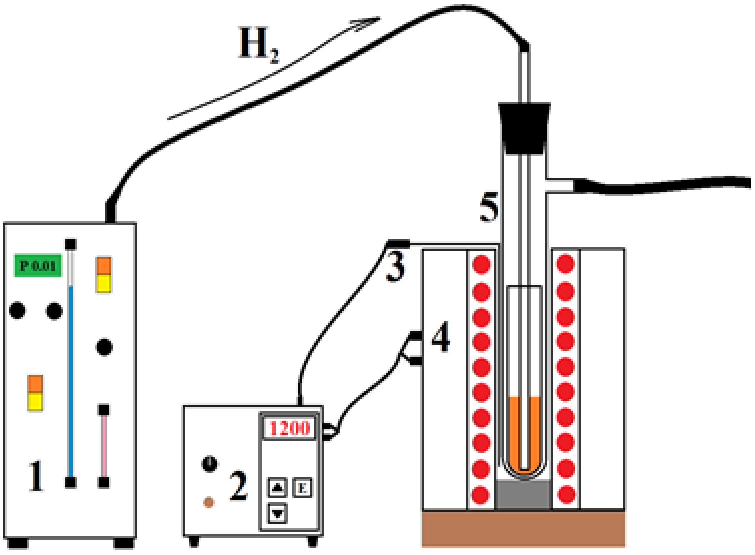

Figure S2. Installation scheme of processing substances in a stream of hydrogen: 1 - a hydrogen generator; 2 - power control unit of electricity supplied to the furnace; 3 – Thermocouple; 4 - electric heating furnace; 5 - reactor with the processed substance
